# Compatibility Enhancement of the Polylactic Acid/Polystyrene Immiscible Blend Using Reactive Graphene

**DOI:** 10.1021/acsomega.5c00440

**Published:** 2025-03-17

**Authors:** Marcos Fernando Perez-Pucheta, Stephen C. Boothroyd, Selene Munoz-Vargas, Karl S. Coleman

**Affiliations:** † Department of Chemistry, Durham University, South Road, Durham DH1 3LE, U.K.; ‡ Department of Chemistry, School of Physical Sciences, University of Liverpool, Peach Street, Liverpool L69 7ZE, U.K.

## Abstract

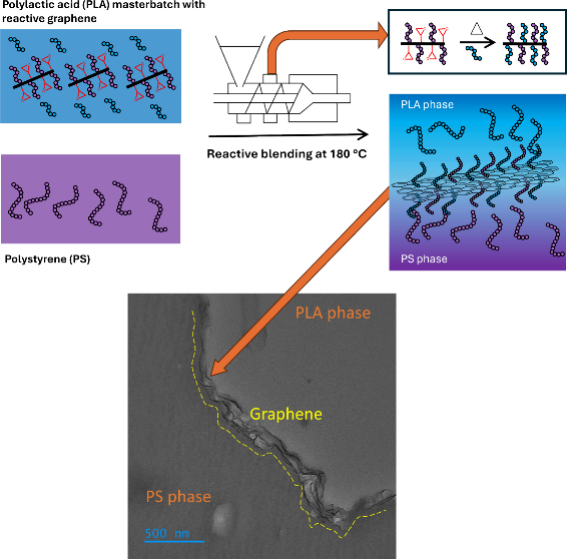

Graphene can be utilized as an additive to produce composites with outstanding properties by improving the stabilization of the morphology of bicontinuous blends. The bicontinuous structure can be used to create novel porous materials and reduce the percolation threshold when graphene is selectively localized at the interface or preferentially in a single phase. This study explores the effect of reactive graphene on the immiscible polystyrene/polylactic acid (PS/PLA) system. Transmission electron microscopy and scanning electron microscopy observations showed that the reactive graphene can be selectively localized at the interface of the PS/PLA system, stopping the coarsening of the structure during static annealing and stabilizing the morphology. Dynamic rheological measurements showed that the addition of reactive graphene had a significant effect on the elastic properties of the blend. Moreover, it was shown that reactive graphene can form a percolated conductive network, which also showed a significant recovery upon destroying the initial microstructure.

## 1 Introduction

Extrusion of polymer blends is a common way to afford materials with unique properties by combining the properties of the homopolymers.^1^ However, most polymer combinations used are immiscible and show phase separation instead, leading to weak interfacial adhesion.^2^ An intuitive way to enhance the compatibility of polymer blends is by using copolymers, which are formed by segments of two homopolymers. The compatibilization effect comes from the reduction of the interfacial tension by the copolymer interfacial localization.^3,4^ However, when premade copolymers are incorporated into the blend, they aggregate and form micelles instead of localizing at the interface.^5,6^ Furthermore, during processing, copolymers already localized at the interface can be pulled out from it and form micelles due to the high shear forces exerted during the extrusion.^7^

An alternative to the use of copolymers is the use of nanofillers such as silica, graphene-based materials, or carbon nanotubes, among others.^8−10^ During polymer blending, nanoparticles are transported because of the internal shear forces and the difference in the surface energy of the particles. From a thermodynamic point of view, when nanoparticles are at the interface of two immiscible phases, the energy of adsorption (*E*_ads_) is considerably higher than the thermal energy *k*_b_*T* and scales with the square of the size of the nanoparticle.^11^ However, the selective interfacial localization of nanoparticles can only be possible during the migration from one phase to the other, so the nanoparticles are kinetically trapped.^8^ Macosko and co-workers showed that, by mixing for more than one minute a polylactic acid (PLA)/polystyrene (PS) blend, the graphene nanoplatelets interfacially trapped migrated to the PS phase. Later research within the same group showed that the same conditions did not work in a PLA/ethyl vinyl acetate (EVA) blend,^12^ highlighting the need for alternatives independent of the mixing time.

An alternative to improve the miscibility of blends is through the interfacial localization of nanoparticles during reactive extrusion.^13^ For example, it has been shown that carbon nanotubes (CNTs) with reactive epoxide groups and long poly methyl methacrylate (PMMA) tails act as a thermodynamic compatibilizer for immiscible polyvinylidene fluoride/poly l-lactide (PVDF/PLLA) blends.^14^ It was proposed that reactive extrusion induced a reaction between the carboxylic functionalities of PLA and the epoxy functionalities of the CNTs, while the miscibility of PVDF and PMMA enhanced CNT dispersion at the interface.^14^ This approach has also been used with other nanoparticles, such as silica,^15^ gibbsite platelets,^16^ and boehmite nanorods,^17^ to improve the compatibility of PVDF/PLA, PLA/polybutylene succinate (PBSU) and PVDF/PLA blends, respectively.

**Figure 1:**
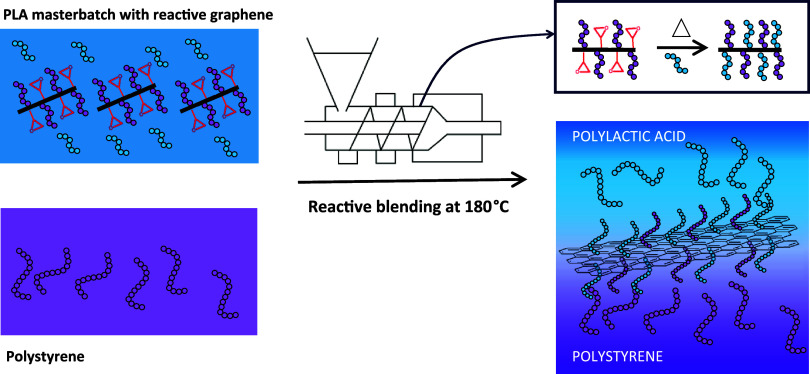
Schematic representation of the mixing process of the polystyrene (PS) and the poly(lactic acid) (PLA) masterbatch containing the reactive graphene.

In this work, we explore how graphene with epoxide groups and pregrafted PS chains can arrest the coarsening of the morphology of the bicontinuous blend PS/PLA. Here, a PLA masterbatch containing the reactive graphene was prepared and mixed with PS in a mini extruder. The process is illustrated in Figure 1. It is expected that during extrusion, the epoxide groups will react with the carboxylic end groups of PLA. Also, pregrafted PS chains will promote entanglement with PS chains from the blend once the reactive graphene reaches the interface. Localization of reactive graphene and its effect on the phase coarsening was directly observed by transmission electron microscopy and scanning electron microscopy. The effect reactive graphene had on the microstructure was measured by dynamic rheological measurements, and the ability of the network to recover upon destruction was tested using rheoimpedance measurements.

## 2 Results and Discussion

### 2.1 Characterization of the Reactive Graphene

**Scheme 1:**
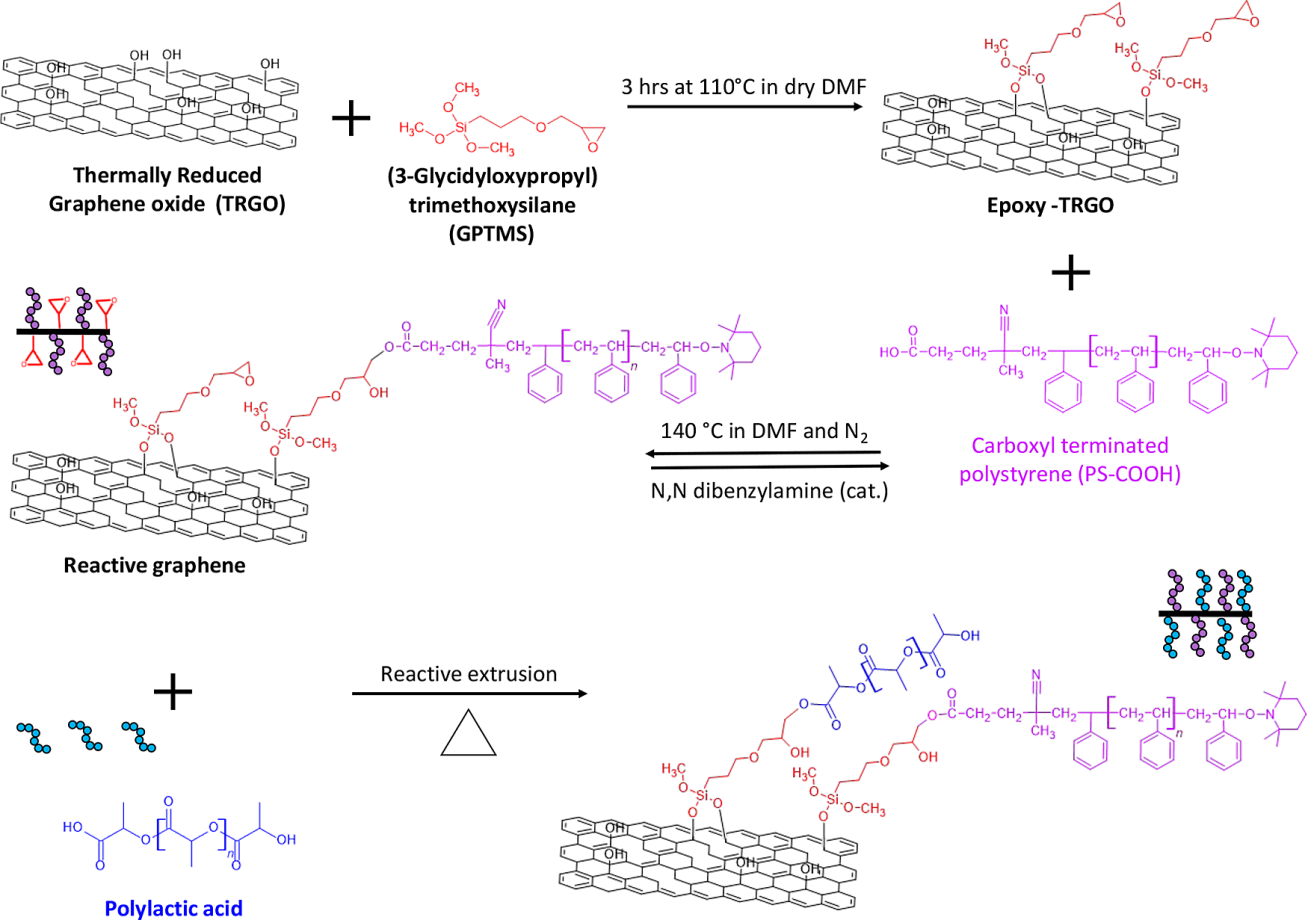
Illustrative Representation of the Synthesis Pathway for Epoxy-Functionalized Thermally Reduced Graphene (Epoxy-TRGO), Reactive Graphene, and Its Interaction with Polylactic Acid Chains during Reactive Extrusion

**Figure 2:**
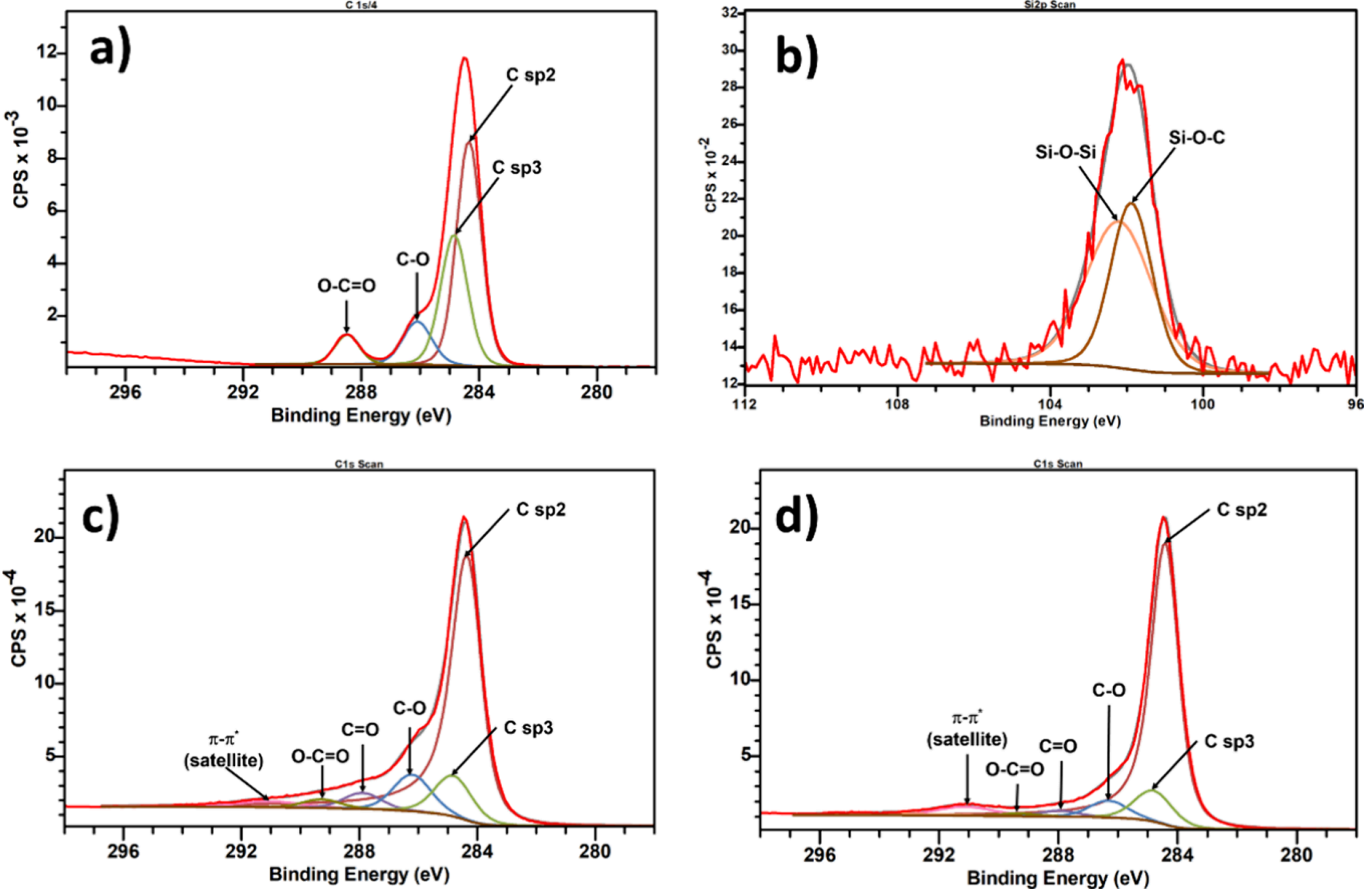
XPS spectra: (a) C 1s of TRGO, (b) Si 2p of Epoxy-TRGO, (c) C 1s of Epoxy-TRGO, and (d) C 1s Reactive graphene.

To synthesize the reactive graphene, graphene oxide was prepared using a modified Hummers method^18^ and thermally reduced (SeeSection 4.3). After reduction, the resulting thermally reduced graphene (TRGO in Scheme 1) exhibited remaining hydroxyl groups on the surface, as shown by XPS analysis in the C 1s (Figure 2a), which were used to introduce epoxide groups by grafting 3-glycidyloxypropyl trimethoxysilane (GPTMS), as shown in Scheme 1. The XPS analysis in the Si 2p region of the epoxide functionalized TRGO, referred to as Epoxy-TRGO is shown in Figure 2b. The assignment of the Si 2p agreed with that of GPTMS grafted onto multiwall carbon nanotubes (MWCNTs).^19^ The peak at 101.8 eV is assigned to Si–O–C from the bond formed from hydroxyl groups on the surface of TRGO and GPTMS, while the peak at 102.2 eV corresponds to siloxane (Si–O–Si) following partial hydrolysis, due to residual water on the TRGO following washing.^19^ Reactive graphene was produced by introducing carboxyl-terminated polystyrene chains (PS-COOH) through a ring-opening reaction, as shown in Scheme 1. A decrease in the proportion of C–O, C=O, and O–C=O groups was observed as each PS-COOH chain grafted introduced additional carbon-containing moieties and a minimal amount of oxygen (Figure 2c,d, respectively). Grafting of PS-COOH is further supported by zeta potential measurements as given in Supporting Information S1.

**Figure 3:**
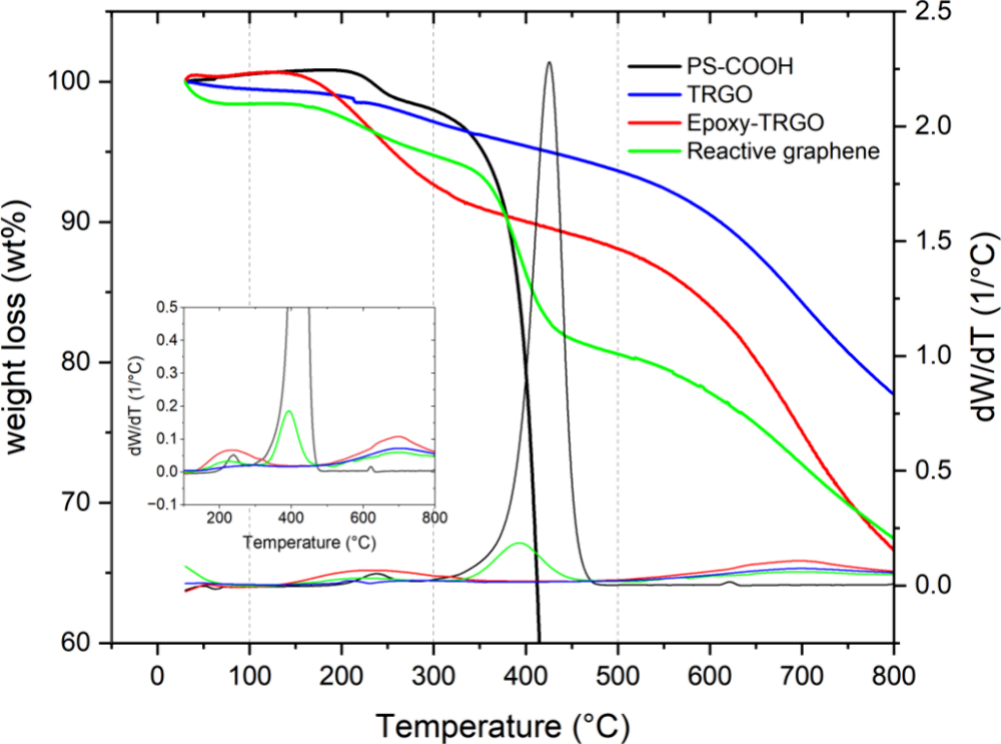
Thermogravimetric analysis from the decomposition of PS-COOH, TRGO, epoxy-TRGO, and reactive graphene; inset: magnification of the first derivative data.

XPS results are supported by thermal gravimetric analysis data. Figure 3 shows the thermograms and the first derivative of the data from TRGO, Epoxy-TRGO, and Epoxy-TRGO-PS (reactive graphene). Three decomposition regions are identified: (1) 100–300 °C, (2) 300–500 °C, and (3) 500–800 °C. In the first region, no significant change occurred for the TRGO, but for the Epoxy-TRGO, there is a 7.6 wt % loss at 300 °C suggesting the loss of more labile groups coming from the GPTMS. In the second region, PS-COOH shows a pronounced weight loss at 426 °C in Figure 3 inset, and as expected, reactive graphene has a peak in this region (392.6 °C), confirming the grafting of the PS-COOH chains. This observation is further corroborated by the similarity in the percentage of remaining weight for both Epoxy-TRGO and Epoxy-TRGO-PS composites (66.6 and 67.4 wt %, respectively). This similarity is likely a consequence of the complete decomposition of the PS polymer chains.

### 2.2 Stability of the Microstructure under Static Annealing

**Figure 4:**
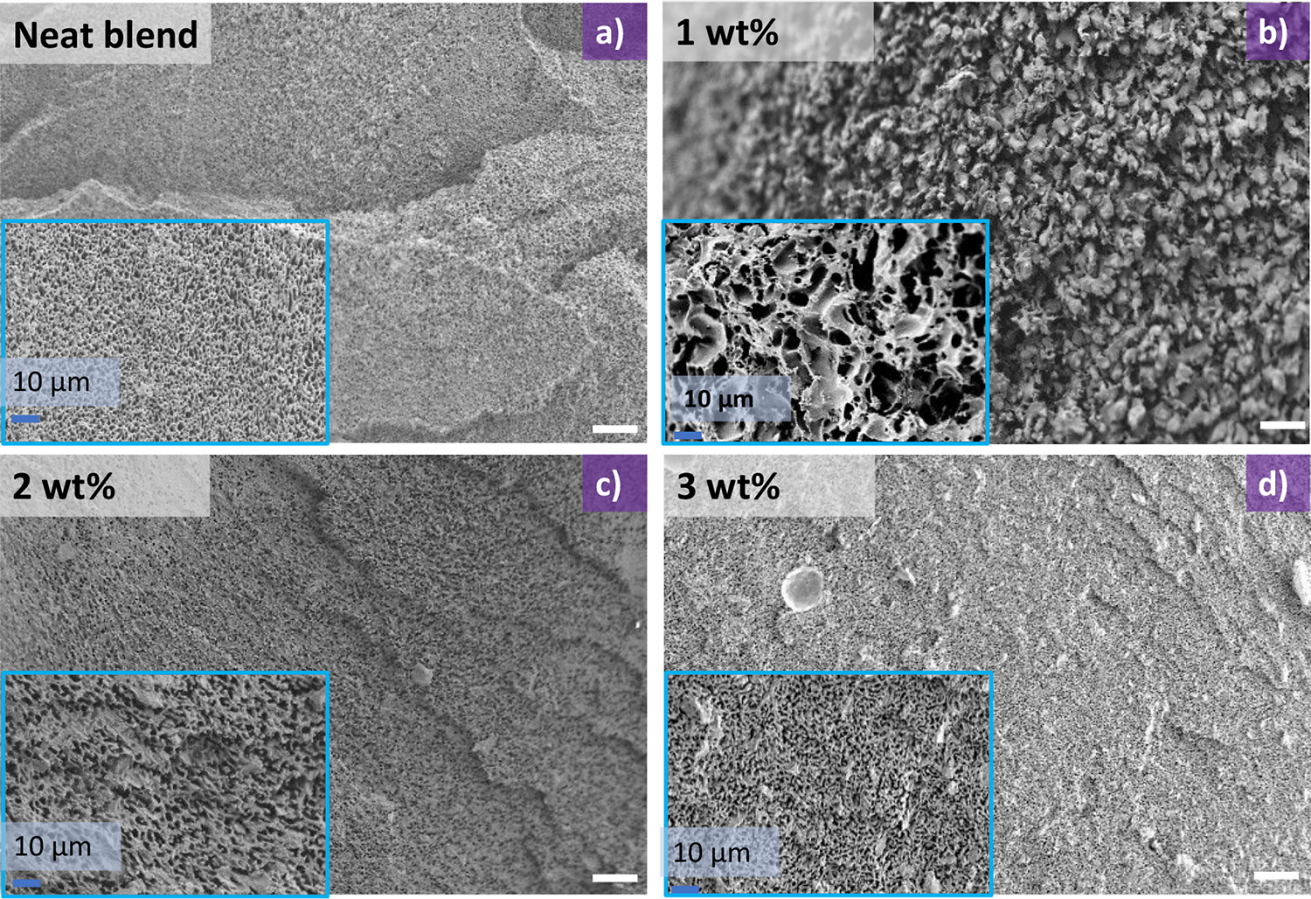
SEM images showing the morphology of neat blend (a) and the filled blends using different loadings of the reactive graphene: (b) 1 wt %, (c) 2 wt %, and (d) 3 wt % immediately after extrusion. To preserve the morphology, samples were quenched in liquid nitrogen and the polystyrene phase was etched in cyclohexane. White scale bars are 40 μm.

**Figure 5:**
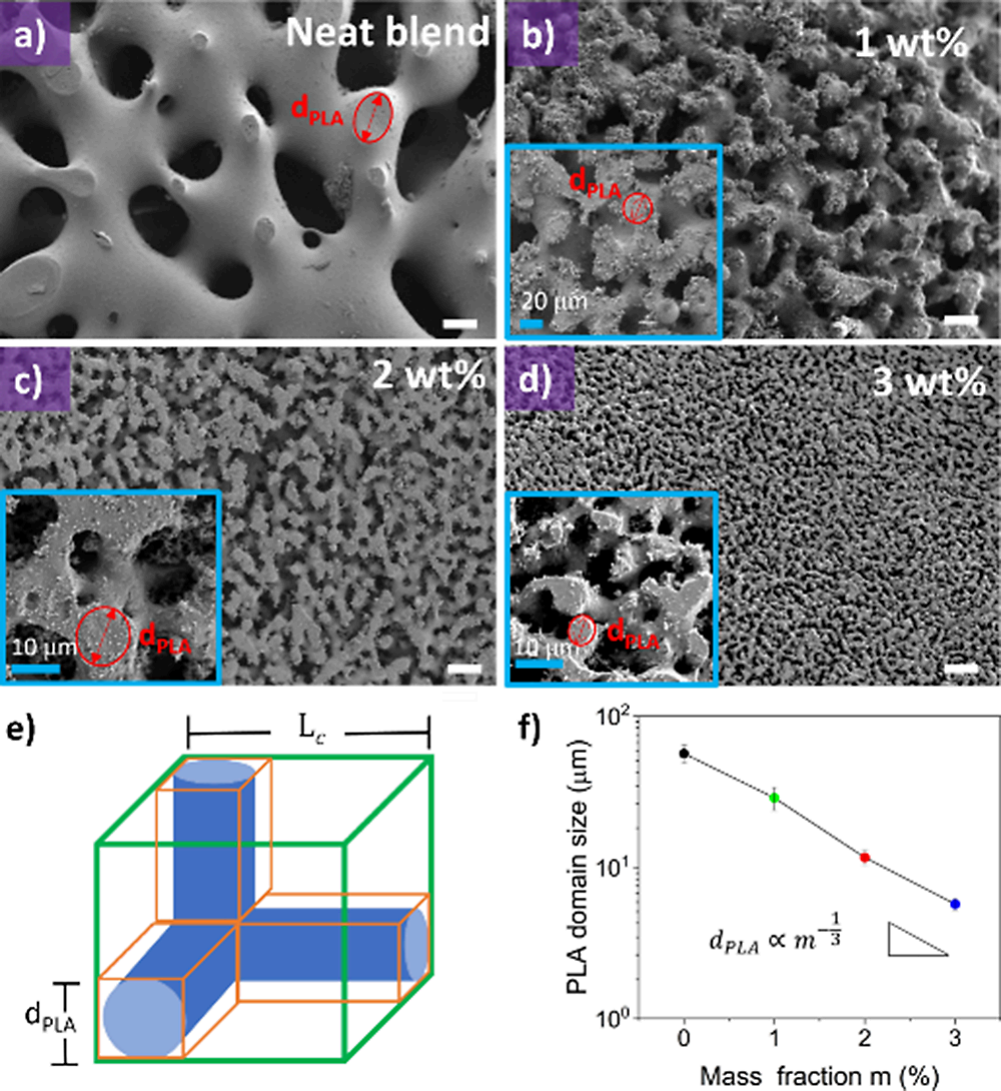
SEM images showing microstructure of the polylactic acid/polystyrene blend, after 30 min annealing at 180 °C and etching the polystyrene phase, using different loadings of reactive graphene: (a) neat blend, (b) 1 wt %, (c) 2 wt %, and (d) 3 wt %. White scale bar is 40 μm in all images. In images c–f, the red circle shows the characteristic length *d*_PLA_ used for measurements. (e) Simplest element constituting a bicontinuous blend adapted from the model proposed by Yu et al.^20^ (f) Dependence of the characteristic length *d*_PLA_ with the loading of reactive graphene.

In brief, a PLA masterbatch containing graphene was mixed in a mini extruder for 10 min at 180 °C (See Section 4 for a full description). Thorough sample mixing is crucial to observing microstructure changes. Therefore, immediately after extrusion, samples were quenched in liquid nitrogen to preserve the initial microstructure, followed by etching of the polystyrene phase in cyclohexanone. The initial microstructures of the neat blend and blends with varying reactive graphene loadings (1, 2, and 3 wt %) are shown to be uniformly mixed (Figure 4a–d). From Figure 5a–d, a coarsening of the blend is observed after 30 min under static annealing, exhibiting a bicontinuous morphology. Figure 5e presents the most basic element of this morphology, as described by Yu et al.,^20^ which is observed in Figure 5a and the insets of Figure 5b–d. From Figure 5f, it is observed that smaller domains of the *d*_PLA_ correspond to a structure with a finer morphology (i.e., smaller *d*_PLA_), and therefore a smaller characteristic length (*L*_c_) of the basic element. Hence, *d*_PLA_ can be linked to the coarsening process. Figure 5f also shows that by increasing the amount of reactive graphene, *d*_PLA_ is reduced following a power law dependence, which is in contrast with the near linear decrease found in other work.^15^ For comparison, for the neat blend *d*_PLA_ = 58.5 ± 7.8 μm and by adding 3 wt % of reactive graphene *d*_PLA_ = 5.7 ± 0.5 μm, which is a decrease in the *d*_PLA_ of around ten times. Consequently, the presence of reactive graphene leads to an increase in the interfacial area, suggesting an enhancement of the compatibility of the blend.

**Figure 6:**
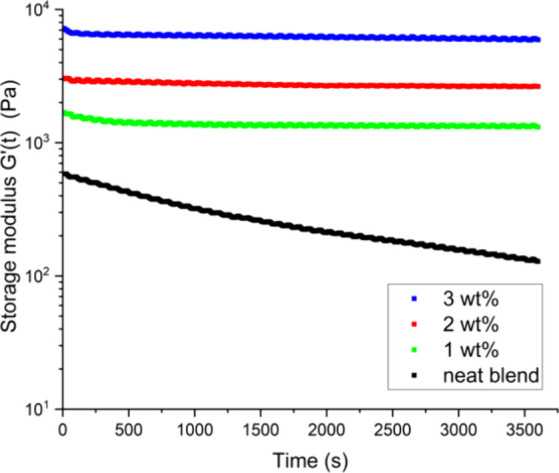
Storage modulus *G*′ evolution in an annealing experiment at 180 °C using a 25 mm parallel plate setup at 1 rad/s.

Based on our previous observations, we investigated the stability of the microstructure by monitoring the evolution of the elastic modulus (*G*′_PLA/PS/graphene_) in a time oscillation experiment (Figure 6). It was suggested by Lee et al. that the storage modulus of an immiscible bicontinuous blend can be decomposed into the contribution from the components of the blend and the interface (*G*′_interface_).^21^ For a fixed frequency, the contribution from the components remains constant in a time oscillation experiment, but the interfacial contribution will decrease asymptotically because of the phase separation.^21,22^ Since *G*′_interface_ is directly proportional to the interfacial area, a continuous decrease in the elastic modulus of the blend (*G*′_PLA/PS/graphene_) is expected because of the reduction in the interfacial area during the phase separation of the blend. In contrast, samples filled with reactive graphene reached a plateau, suggesting that the morphology stopped evolving, as has previously been reported.^22^

### 2.3 Formation of an Interfacial Percolated Network

**Figure 7:**
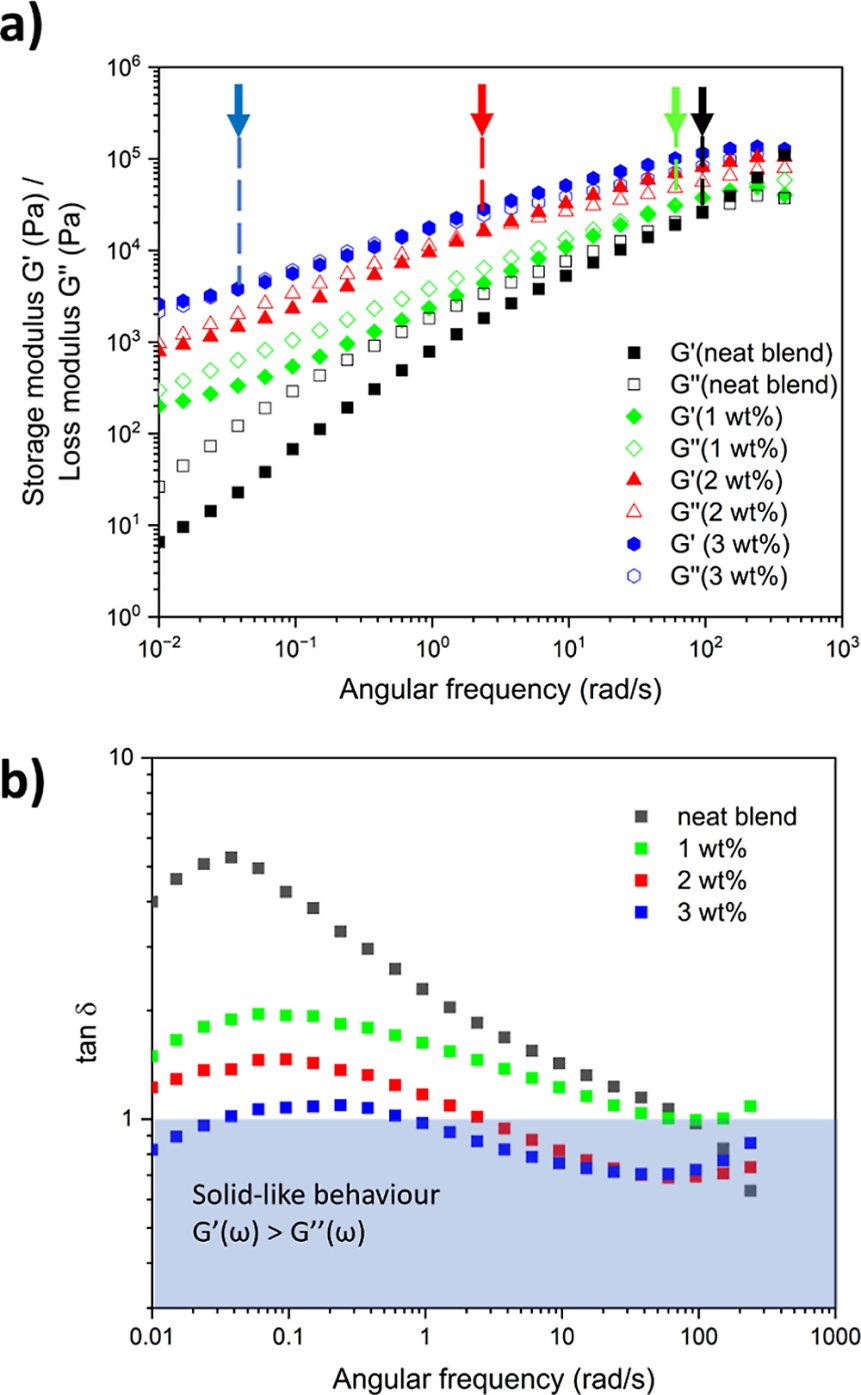
(a) Frequency sweep at 180 °C of *G′*(ω) and *G″*(ω), the arrows indicate the cross over frequency point, that is when *G′*(ω) = *G″*(ω), and (b) tan δ (ω).

Rheological frequency sweeps show a reduction in the terminal slope and the eventual appearance of a plateau at low frequencies due to the formation of elastic networks in polymer matrices.^8,9,12,14,23−25^ This behavior is observed in Figure 7a for the compatibilised blends, becoming more predominant as the amount of reactive graphene increases.^8^ Moreover, a shift of the crossover frequency *G*′(ω) = *G*″(ω), is observed toward lower values, meaning the network is elastic over longer time scales. This is more evident from the tan δ graph (Figure 7b) where the sample containing 3 wt % of reactive shows predominantly elastic behavior (tan δ < 1), but even the use of 1 wt % of reactive graphene dramatically improved the elastic response, which is in contrast with the neat blend that exhibits a predominantly viscous behavior over almost the whole frequency sweep (tan δ > 1). Similarly, data from dynamic mechanical analysis (see Figure S1) revealed improvements in the elastic response, consistent with enhanced mechanical properties.

Figure 7a shows an increase in the storage modulus over the whole frequency range for the filled samples, which is more pronounced in the low-frequency range. In a bicontinuous blend, the interfacial contribution dominates the component contributions at low frequencies, so the storage modulus *G*′(ω) approximates the interfacial modulus *G*′(ω)_interfacial_, which is proportional to the interfacial area and inversely proportional to the interfacial tension.^20,26^ Therefore, observations in the low-frequency region suggest that the interfacial localization of reactive graphene has a compatibilizing effect by increasing the interfacial area and reducing the interfacial tension between PS and PLA phases.

### 2.4 Localization of the Reactive Graphene in the Blend

**Figure 8:**
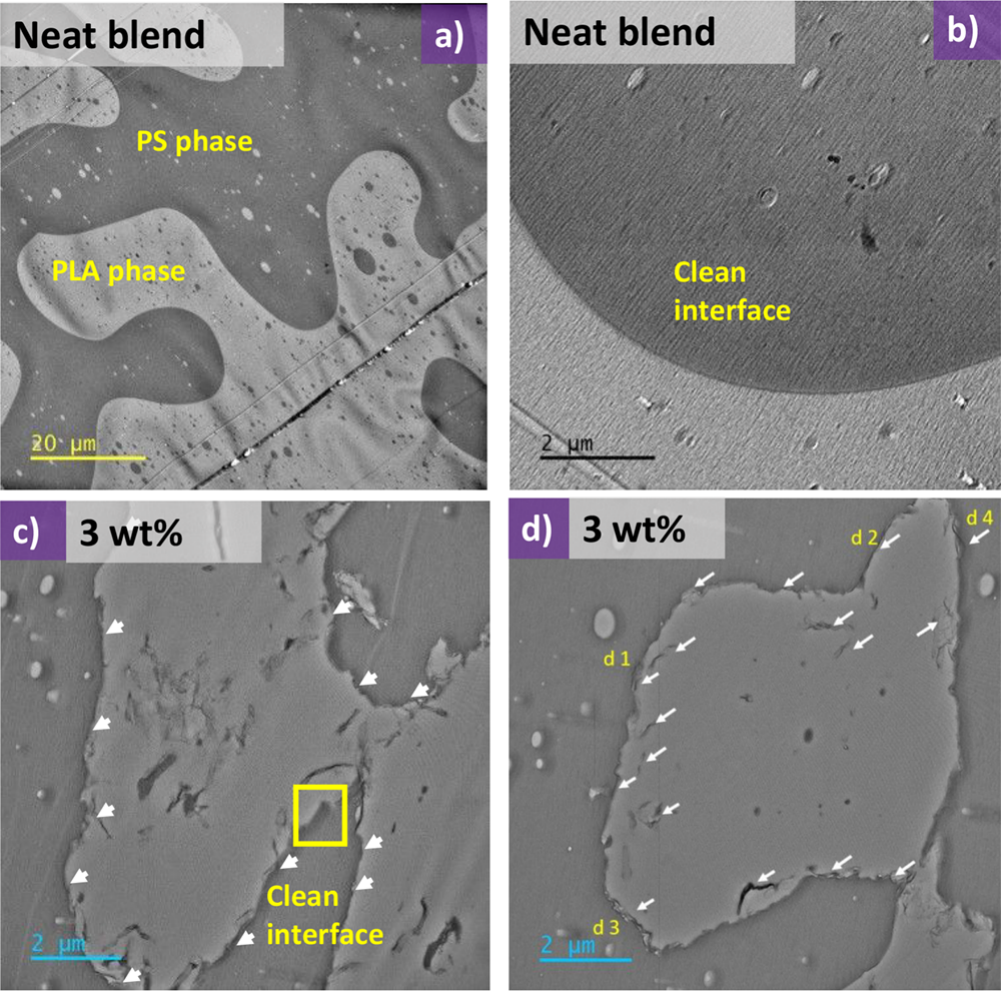
(a, b) TEM images of the neat blend showing a clean interface (scale bar: 20 and 2 μm, respectively). (c, d) Typical images of the blend with 3 wt % reactive graphene. White arrows show reactive graphene trapped at the interface or in the PLA phase (Scale bar: 2 μm).

**Figure 9:**
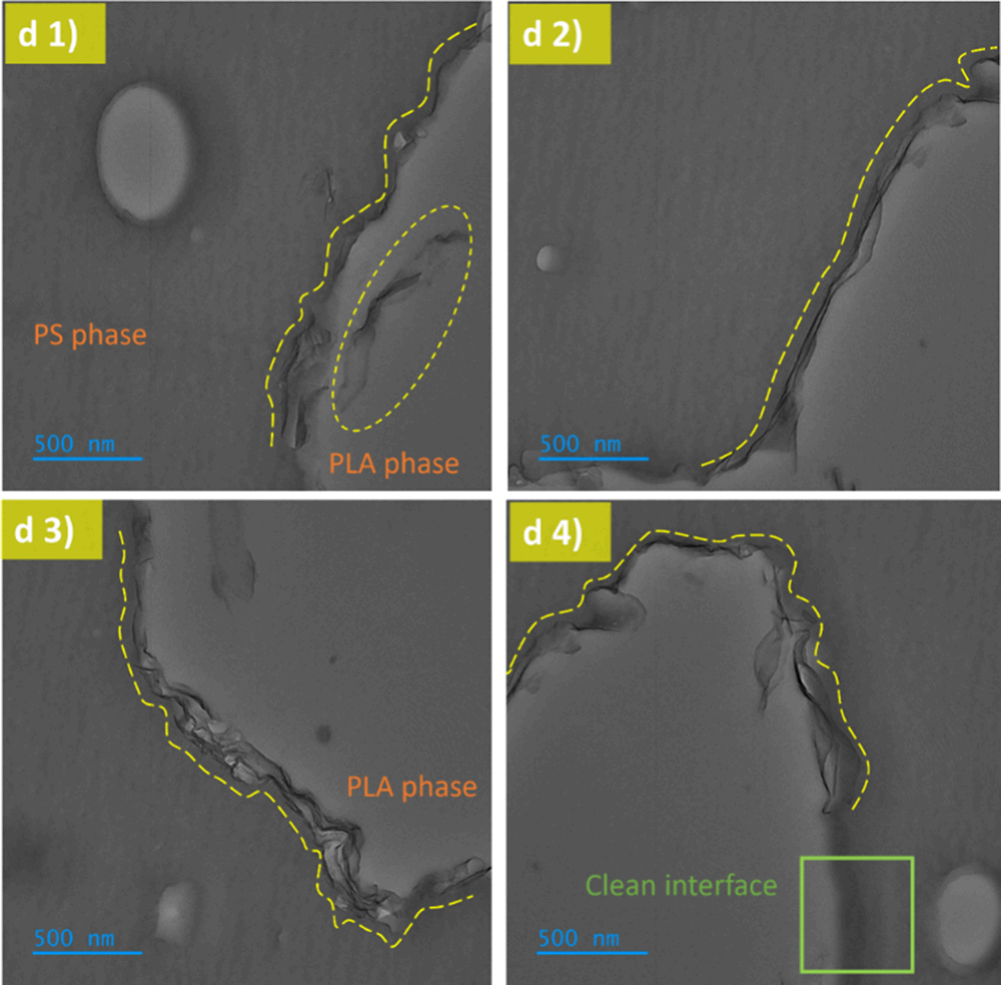
(d1–d4) High magnification TEM images from selected areas shown in Figure 8d demonstrating that the reactive graphene is localized at the PS/PLA interface. Contours of the reactive graphene are highlighted with dashed yellow lines. The green box in (d4) shows a clean area free of reactive graphene.

To further investigate the localization of the reactive graphene, the sample containing 3 wt % was cross-sectioned in an ultramicrotome and imaged in a transmission electron microscope (TEM). PS is known to be resistant to degradation under irradiation, while PLA is susceptible to it. Therefore, the contrast will be induced due to the mass loss.^27^Figure 8a shows the typical image from the neat blend, confirming the bicontinuous nature from SEM observations. Figure 8b illustrates a high-magnification area of the interface that is smooth and free of particles. Figure 8c,d illustrates typical images of the blend with 3 wt % of reactive graphene. The wetting coefficient has been used for predicting the final localization of nanoparticles in immiscible blends^8,28^ and calculations indicate that reactive graphene should predominantly remain in the PLA phase (see Table S2). However, TEM observations reveal a preference for the localization of the reactive graphene at the interface. This is observed in high-magnification images in Figure 9d1–d4, taken from the areas shown in Figure 8d; more TEM images are given in the Supporting Information (Figures S2–S5). These observations also confirm an interfacial network formed by reactive graphene.

### 2.5 Rheoimpedance Observations

**Figure 10:**
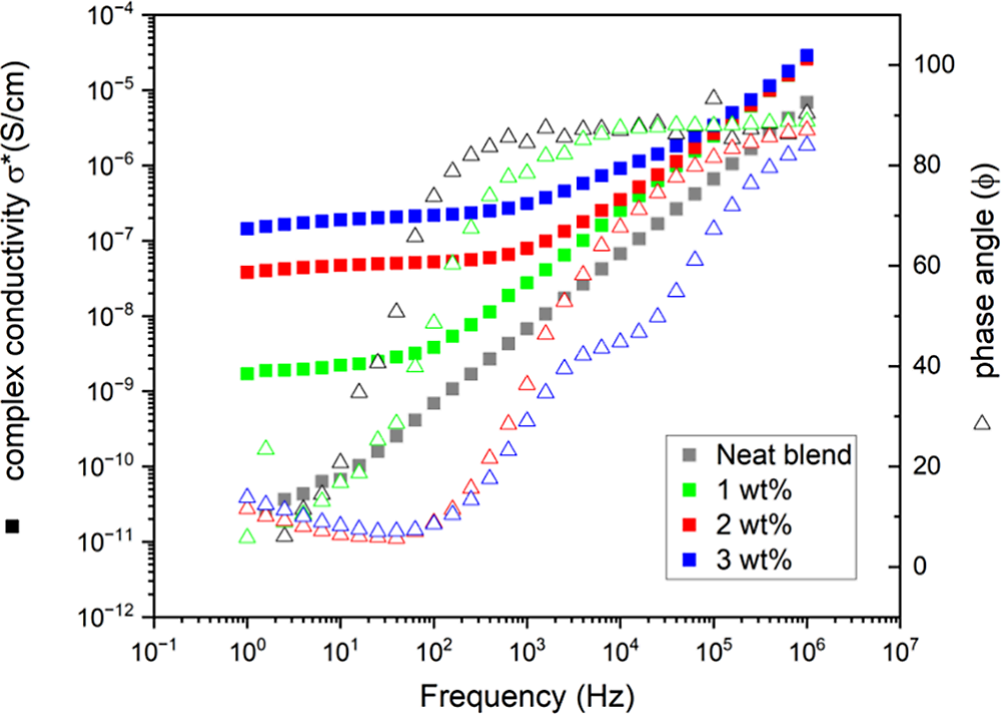
Graph of the frequency-dependent complex conductivity (σ*) and the phase angle (Φ) for different loadings of reactive graphene.

Since graphene platelets in a polymer blend can form electrically conductive paths, impedance measurements can be used to track the evolution of the network formed in the blend. Impedance is the resistance encountered by an alternating current, comprising of the contributions from the real (*Z*_real_) and the imaginary parts (*Z*_imag_). These quantities are related to the complex conductivity through eqs2 and 3.
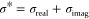
1
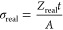
2
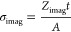
3Where *t* and *A* are the thickness and cross-sectional area of the sample. From Figure 10, it is observed that the complex conductivity of neat blend shows a linear relationship with respect to the frequency, having a slope ∼1 and a phase angle close to 90° which is consistent with a purely capacitive (dielectric) behavior at high frequencies.^29^ In contrast, the filled blends showed a region where the complex conductivity is frequency independent and the phase approximates to zero, which is characteristic of a purely resistive behavior.^9,30^ The resistive character of the network is the result of free electron conduction through the reactive graphene network. Therefore, measurements of the real part of the impedance at low frequencies can provide information about the formation/disruption of the reactive graphene networks. Figure 10 suggests that the sample with 1 wt % of reactive graphene displays a resistive behavior at low frequencies, but this is more pronounced for samples filled with 2 and 3 wt % for which the phase angle remains close to zero over a broader frequency range. Moreover, the sharp increase in the complex conductivity at low frequencies from 1 to 2 wt % and the broader frequency region where the phase angle is close to zero indicate the formation of a percolated network. A decrease in the impedance can also be observed in the Nyquist plots shown in Figure S6.

**Figure 11:**
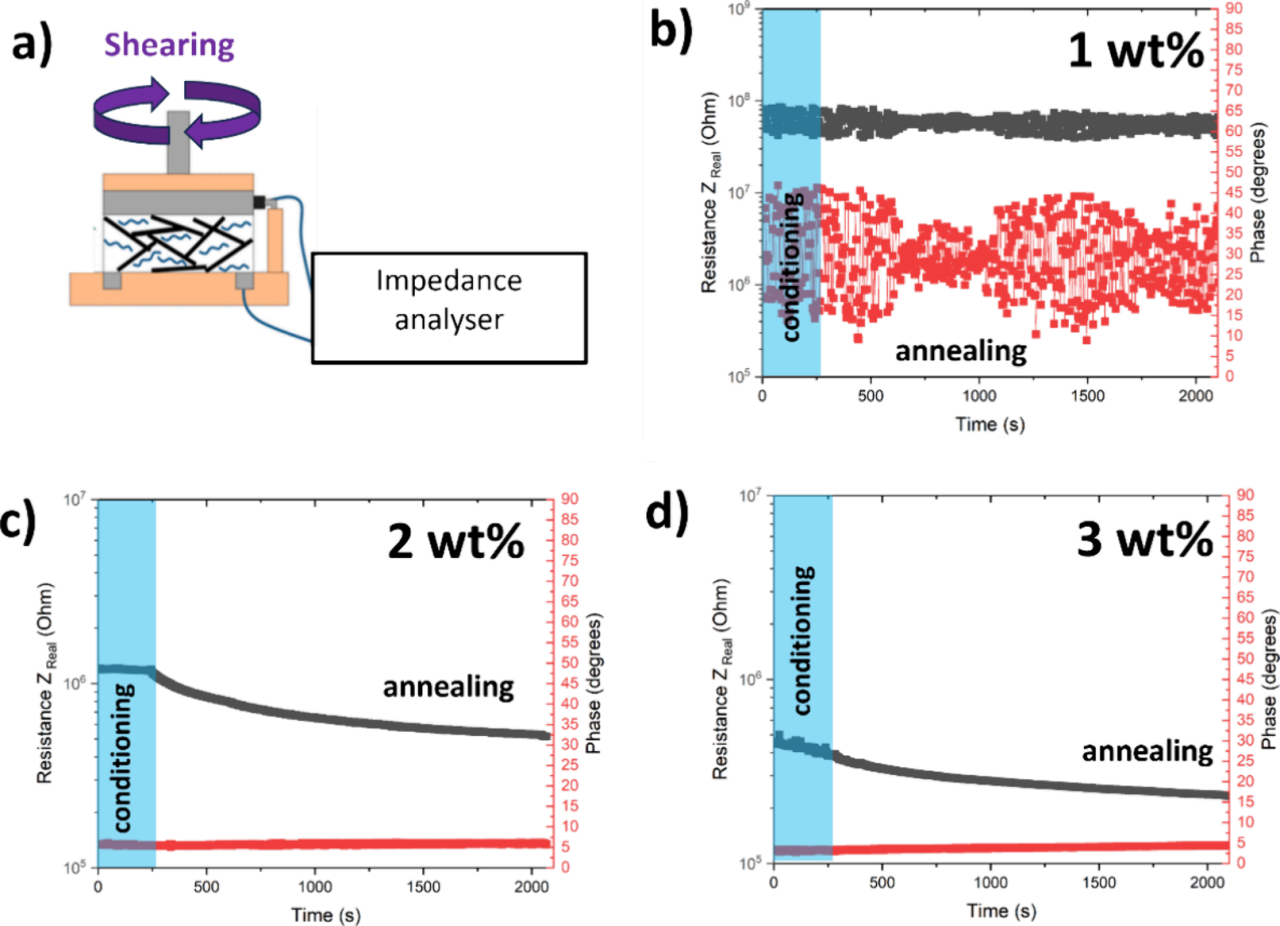
(a) Experimental setup adapted from ref (31). (b–d) Rheoimpedance response recorded at 20 Hz and 180 °C at different loadings (1, 2, and 3 wt %). Conditioning step: shearing at 0.01 s^–1^ and 180 °C for 5 min, followed by static annealing. Black points correspond to the real part of the resistivity and the red points correspond to the phase shift.

In this work, we used a rheometer coupled to an electrical impedance analyzer (Figure 11a) to control the temperature and the shear rate for sample conditioning while monitoring the impedance over time. Figure 11b shows the 1 wt % sample has an incomplete network causing significant phase angle oscillation and a high final impedance (4.3 × 10^8^ Ω). In contrast, the resistivity of the 2 wt % blend was almost 3 orders of magnitude lower (2.3 × 10^5^ Ω), and its phase angle remained low at around 5 degrees and stable throughout the experiment (Figure 11c). Upon increasing the load to 3 wt % both the phase and the resistivity exhibited minimal changes compared to the 2 wt % sample (Figure 11d). From the percolation theory perspective, 2 wt % of reactive graphene is the critical value for network formation.

**Figure 12:**
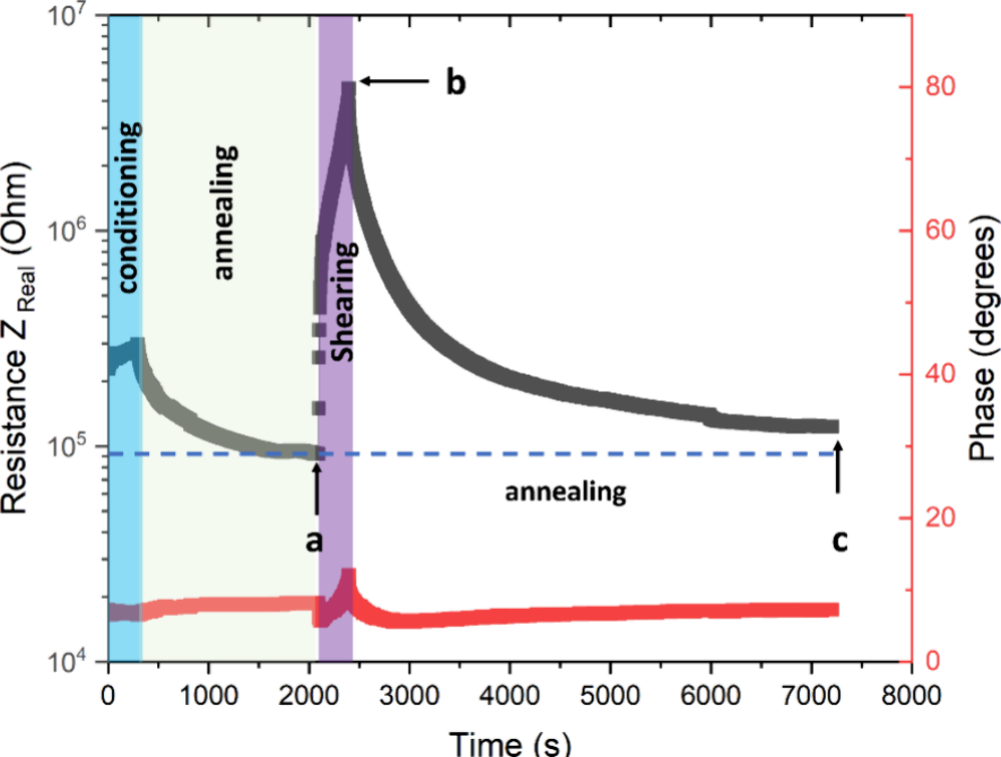
Rheoimpedance response recorded at 20 Hz and 180 °C. Conditioning step: shearing at 0.01 s^–1^ and 180 °C. Shearing step: 0.1 s^–1^ for 5 min followed by static annealing. Black points correspond to the real part of the resistivity and the red points correspond to the phase shift.

The ability of the graphene network to recover once the microstructure has been disrupted was assessed. First, the sample was conditioned by applying shear at a very low speed (0.01 s^–1^) followed by an annealing step to allow the morphology to reach equilibrium. This was followed by a high shear step (0.1 s^–1^) to disrupt the equilibrium morphology (Path a→b in Figure 12). Upon disruption of the morphology, a sharp increase in the resistance is observed, while the phase angle shows only a minor increase. This is the result of the combined effect of the disruption of the network and the alignment of the trapped interfacial graphene with the shear.^31^ Once the shearing ceased, the sample was subjected to static annealing (Path b→c in Figure 12), and an immediate decrease in the resistance was observed, suggesting that the disrupted network was reforming. At the end of the experiment, the network recovered 74.5% of the resistivity with respect to the value obtained after the annealing step. This demonstrates the presence of a robust interconnected network that is resistant to changes in the microstructure.

### 2.6 Mechanism

**Figure 13:**
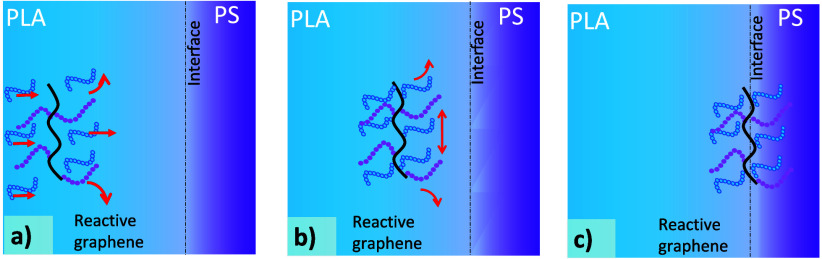
Schematic representation of the localization of reactive graphene. (a) Remaining epoxide groups react with carboxyl groups on PLA chains aided by high shear forces. Those forces also transport reactive graphene by convection to a zone near the interface. (b) Film drainage of reactive graphene driven by surface energy difference. (c) Graphene is localized at the interface.

The proposed mechanism, as shown in Figure 13a–c, describes the two stages through which migration from the PLA phase to the interface is thought to occur. First, the remaining epoxide groups on the reactive graphene react with terminal carboxyl groups from the PLA phase during the reactive extrusion (Scheme 1), as also suggested in previous studies.^32−41^ This reaction happens simultaneously with the transport of the reactive graphene to the interface. The high shear forces during extrusion cause the reactive graphene to align parallel to the interface, as shown by Boothroyd et al.^31^ In the second stage, the difference in the surface tension of the two polymers and the difference in normal stresses between the two polymers caused by the shear forces push the reactive graphene to the interface.^42^ Once the reactive graphene is located at the interface, the attractive forces exerted by the PS phase on the grafted PS chains are compensated for by those of the PLA phase on the grafted PLA chains, causing the reactive graphene to remain there. We found that even when a long mixing time was used (10 min), no reactive graphene was found in the PS phase (Figures S2–S5). This is in contrast with previous studies where the migration of nonfunctionalized carbon black, carbon nanotubes, and graphene nanoplatelets, in different immiscible blends, occurs during the first few minutes of blending.^10,28,43−46^ Furthermore, our surface energy calculations (see Supporting Information S2) indicated that the reactive graphene should reside in the PLA phase only. However, our observations show it is predominantly located at the interface even when processing for a long time and after a static annealing process, suggesting a high energy of adhesion of the reactive graphene to the interface.

## 3 Conclusions

Immiscible blends form interpenetrated networks that can be used as a scaffold for nanoparticles to be selectively localized in a single phase or at the interface. The nanoparticles are usually distributed in a single phase; however, research has indicated that the use of Janus nanoparticles allows for selective localization at the interface. This enhances interfacial strength, improves miscibility, and forms an electrically conductive network, as demonstrated in PVDF/PLLA blends.^14,15^ However, there are very few reports on the localization of graphene-based platelets at the interface, with most systems reporting the segregation in a single phase.^9,12,46^ Here, we have used epoxide and polystyrene-grafted reduced graphene oxide, incorporated in a PLA masterbatch, extruded with PS to form a stable bicontinuous blend with a percolated electrical pathway. Rheological data suggest the formation of a stable network formed by the interfacially localized reactive graphene that decreases the interfacial tension between PLA and PS, consistent with the increase in interfacial area, as shown by a 10-fold decrease in PLA domain size in SEM images with 3 wt % reactive graphene platelets. TEM observations confirmed that the graphene platelets remained localized at the interface. Based on our observations, we propose a mechanism where the high shear forces during the extrusion drag the reactive graphene close to the interface while promoting the reaction between the remaining epoxide groups in the reactive graphene and the carboxyl groups from PLA chains. The attached PLA and PS chains on the graphene surface enhance interfacial adhesion because of their Janus nature. Rheoimpedance experiments corroborate this, demonstrating that a stable, conductive network forms with the addition of at least 2 wt % reactive graphene, one that resists shear and nearly fully recovers its initial resistivity upon cessation of shear. Our results showed the use of graphene for the compatibilization of immiscible blends as an alternative method for the development of advanced materials with improved rheological and electrical properties, for a range of applications such as smart fibers and environmental sensors.^47,48^

## 4 Methods

### 4.1 Materials

All chemicals were used without further purification. Sulfuric acid (98%, Fisher Scientific), phosphoric acid (85%, Fisher Scientific), hydrochloric acid (37%, Fischer Scientific), hydrogen peroxide (37%, Sigma-Aldrich), graphite flakes (99%, 325 mesh, Sigma-Aldrich), (3-glycidyloxypropyl)trimethoxysilane (98% Sigma-Aldrich), dry *N*,*N*-dimethylformamide (99.8% Fisher Scientific), styrene (99.9% Sigma-Aldrich), 2,2,6,6-tetramethyl-1-piperidinyloxy (TEMPO 98%, Sigma-Aldrich), 4,4′-azobis(4-cyanovaleric acid) (98% Sigma-Aldrich), methanol (AR for analysis, > 99.8%), dibenzylamine (97% Sigma-Aldrich), polystyrene (*M*_w_ ∼ 192,000, Sigma-Aldrich), and polylactic acid (*M*_w_ ∼ 230,000, GoodFellow).

### 4.2 Synthesis of Carboxyl Ended Polystyrene

The method described here was adapted from the literature.^49^ 20 mL of styrene were passed through a silica column to remove stabilizers, and 8 mL was collected and transferred to a Schlenk tube with a stir bar. Then, 45.3 mg of TEMPO and 81.3 mg of ACVA were added to the tube. The mixture was frozen and the vessel evacuated, and then it was left to thaw. The freeze–pump–thaw cycle was repeated three times, and in the last cycle, the vessel was refilled with argon. The tube was then transferred into an oil bath preheated at 135 °C and the reaction was allowed to proceed for 1 h, after which the tube was placed into a cool water bath and opened to the atmosphere to quench the reaction. The synthesized polymer was precipitated in 200 mL of methanol and vacuum filtered over a nylon membrane (0.2 μm pore size, Fisherbrand). The synthesized polymer was washed three more times with 50 mL of methanol. The residue was then collected in a sample vial and dried under vacuum (*p* < 20 mbar) for 12 h before use.

### 4.3 Synthesis of Graphene Oxide and Preparation of Thermally Reduced Graphene Oxide

Graphene oxide preparation: The procedure for the synthesis of graphene oxide has been previously reported in the literature.^18^ Typically, a 9:1 mixture of concentrated H_2_SO_4_/H_3_PO_4_ (360:40 mL) was added to 3 g of graphite flakes and stirred for 2 min a three-neck round-bottom flask. Then, 18 g of KMnO_4_ was added slowly. After stirring for 30 min at room temperature, the mixture was immersed in an oil bath. The temperature was increased in steps of 2 °C/5 min to avoid overheating. After reaching 58–60 °C, the reaction mixture was allowed to react for 12 h. Then, the reaction was cooled to room temperature and poured onto 400 mL of ice and 8 mL of H_2_O_2_ (37% vol/vol) and left until it reached room temperature. The mixture was centrifuged at 4000 rpm for 30 min and repeated twice, adding 500 mL of deionized water and 5 mL of H_2_O_2_ (37% vol/vol) each time and stirring for 30 min before centrifuging. The sample was treated with a 10 vol % HCl solution and passed through a cross-flow filtration system at 50 mL/min. The workup procedure finished once the pH from the permeate was ∼6.5 to 7.0.

Thermally reduced graphene oxide preparation: Around 50 mg of freeze-dried graphene oxide was introduced in the center of a quartz tube. The tube was placed in a tube furnace (MTI OTFX12000) and flushed with 20 mL/min of argon for 10 min to remove the remaining air. The heating profile was as follows: (1) ramp from room temperature to 400 °C at 19 °C/min, (2) dwell at 350 °C for 30 min, and (3) allow to cool to room temperature. The thermally reduced graphene oxide was transferred into a bottle and kept under a vacuum prior to use.

### 4.4 Silanization of Thermally Reduced Graphene Oxide (Epoxy-TRGO)

In a glovebox, 300 mg of thermally reduced graphene oxide was mixed with 9 mL of (3-glycidyloxypropyl)trimethoxysilane and 100 mL of dry *N*,*N*-dimethylformamide. The mixture was stirred for 3 h in a nitrogen atmosphere and then the solution was heated at 110 °C for 3 h. It was left to reach room temperature before filtering over a PTFE membrane (0.2 μm pore size, Merck) and washed with 75 mL of acetone two times more. The residue on the filter was transferred into a sample vial and left to dry under vacuum (*p* < 20 mbar) at 40 °C.

### 4.5 Synthesis of Reactive Graphene (Epoxy-TRGO-PS)

1 g of carboxy terminated polystyrene (PS-COOH) and 1 g of epoxy functionalized graphene (Epoxy-TRGO) were transferred into a round-bottom flask. Then, 350 mL of dry *N*,*N*-dimethylformamide and 1.5 mL of N,N-dibenzylamine as catalyst were added. The mixture was stirred for 30 min at 140 °C for 24 h. The flask was cooled to room temperature before vacuum filtration over a PTFE membrane (0.2 μm pore size, Merck). The filter cake was washed three times with 75 mL of tetrahydrofuran to remove residual polymer. The solid residue was collected and dried under vacuum (*p* < 20 mbar) at 40 °C for further use.

### 4.6 Preparation of the PLA and Reactive Graphene Masterbatch

A 2 mg/mL solution of Epoxy-TRGO-PS in *N*,*N*-dimethylformamide was prepared and mixed for 1 h. Polylactic acid was mixed with *N*,*N*-dimethylformamide in a proportion of 2 mg/mL and mixed at 80 °C for 2 h using a shear mixer (IKA EURO-ST P CV S2). The two solutions were mixed 2 h more and then precipitated in 400 mL of methanol. The polymer precipitated was washed with 200 mL of methanol using vacuum filtration. The polymer masterbatch was dried under vacuum (*p* < 20 mbar) at 40 °C.

### 4.7 Preparation of the Polystyrene with the PLA Masterbatch

The corresponding amounts for 1, 2, and 3 wt % % of the PLA masterbatch were ground using a pestle and mortar until a fine powder was obtained. Then, it was introduced into a mini extruder, HAAKE microconical twin extruder compounder, under a nitrogen atmosphere followed by introducing the polystyrene pellets, it was then mixed for 10 min at 100 rpm. The extrudate was immediately quenched in liquid nitrogen to preserve the morphology. *Note: Above 3 wt % the viscosity of the mixture increases considerably, making extrusion difficult.*

### 4.8 Characterization Methods

#### 4.8.1 Thermogravimetric Analysis

Samples were pressed to form pellets. TGA was conducted using a PerkinElmer TGA 8000 heating system from room temperature to 800 °C at 10 °C/min under either air or an argon flow (30 mL/min).

#### 4.8.2 Sample Preparation of Polymer Samples for SEM Imaging

A scanning electron microscope (Zeiss Gemini 360 VP) was used for imaging. An in-lens detector was used, with an accelerating voltage of 5 kV and a working distance of 6 mm. Sample preparation: A piece of the extruded polymer quenched in liquid nitrogen was placed in a sample vial with cyclohexanone at 50 °C and left overnight to etch the polystyrene phase. The samples were mounted on an aluminum stub and fixed with carbon tape. Samples were sputter coated with 20 nm of Au/Pd to avoid charging effects during imaging.

#### 4.8.3 Sample Preparation of Polymer Samples for TEM Imaging

TEM images were recorded using a JEOL 2100F FEG TEM operating at 200 kV. Sample preparation: A piece of the extruded polymer was quenched in liquid nitrogen, annealed if necessary, and microtomed. These samples were deposited on a copper grid with no supporting carbon layer.

#### 4.8.4 XPS

A Kratos Axis Ultra DLD system was used to collect XPS spectra using a monochromatic Al Kα X-ray source operating at 120 W (10 mA × 12 kV). All data were analyzed using CasaXPS (v2.3.24)^50^ after subtraction of a Shirley background and using modified Wagner sensitivity factors as supplied by the instrument manufacturer. Curve fits were performed using an asymmetric Lorentzian form (LA Lineshape in CasaXPS), whereas the line shape for graphitic, sp^2^ carbon, was based on a clean highly ordered pyrolytic graphite sample.

#### 4.8.5 Rhological Measurements

Rheological measurements were recorded by using a Discovery HR-2 rheometer (TA Instruments). Frequency sweep measurements were done using a strain of 1% at 180 °C. To prepare the samples, around 500 mg of the (PLA-Reactive graphene)/PS was hot pressed using 5 tons at 180 °C in a 25 mm diameter mold and 1 mm thickness. After 7 min, the pressure was released.

#### 4.8.6 Rheoimpedance Experiments

The rheometer used for these measurements was an AR 2000 (TA Instruments). Samples were prepared as described in the previous section. The samples were placed in the rheometer using a setup we have reported previously using the environmental test chamber with a nitrogen atmosphere.^31^ The bottom geometry included a ring electrode with an outer diameter of 25 mm and an inner diameter of 19 mm, creating a more defined shear rate, while the top plate functioned as the sense electrode. The electrical signal was collected by using a potentiostat (Palmsense 4) over the whole experiment. Throughout the experiment, a sinusoidal signal with a frequency of 20 Hz and an amplitude of 0.1 V was applied.

## Supporting Information Available

Zeta potential measurements, estimations of the reactive graphene localization based on wetting coefficient calculations, supporting TEM images, and Nyquist plots from the data presented in Figure 11 (PDF)
