# Supplementary material for: Compatibility Enhancement of the Polylactic Acid/Polystyrene Immiscible Blend Using Reactive Graphene
Source: ACS Omega. 2025 Mar 17;10(12):12615–25. doi: 10.1021/acsomega.5c00440 (PMC11966257; doi:10.1021/acsomega.5c00440)
Supplement: Supplementary file 1 — ao5c00440_si_001.pdf [file ao5c00440_si_001.pdf]

Compatibility enhancement of the polylactic acid/polystyrene immiscible blend using reactive graphene

Marcos Fernando Perez-Pucheta[1†], Stephen C Boothroyd[1‡], Selene Munoz-Vargas[1†] and Karl S Coleman[2*]

[1]Department of Chemistry, Durham University, South Road, Durham DH1 3LE, U.K.

[2]Department of Chemistry, School of Physical Sciences, University of Liverpool, Peach Street, Liverpool L69 7ZE, U.K.

*Corresponding author: karl.coleman@liverpool.ac.uk

†Current address: Department of Chemistry, School of Physical Sciences, University of Liverpool, Peach Street, Liverpool L69 7ZE, U.K.

‡ Current address: Uniliver Materials Innovation Factory, University of Liverpool, 51 Oxford Street, Liverpool, L7 3NY, U.K.

Supporting information

S1. It is widely accepted that zeta potential values represent the colloidal stability as follows: highly stable (> 30 mV), moderately stable (20-30 mV), relatively stable (10-20 mV) and unstable (0-10 mV).[1] Two solvents were chosen: methanol and tetrahydrofuran. Since polystyrene is insoluble in methanol but quite soluble in tetrahydrofuran (THF), it was expected a low zeta potential value in methanol but not in THF. Furthermore, thermally reduced graphene oxide (TRGO) showed low stability corresponding to low zeta potential values both in methanol and THF. However, upon introducing epoxy groups, the stability significantly improved. Zeta potential values are summarized in Table S1.

Table S1 Zeta (ζ) potential measurements of the stability of 1 mg/ml solutions of TRGO, Epoxy-TRGO and Epoxy-TRGO-PS in methanol and tetrahydrofuran.

	ζ potential [mV]	
	Methanol	Tetrahydrofuran
TRGO	16.2	11.8
Epoxy-TRGO	25.1	51.8
Epoxy-TRGO-PS	2.1	39.4

S2. In this work, the surface energy was approximated using thin films of graphene-based nanoparticles deposited on a PTFE membrane as substrates. Two testing liquids were used: water and diiodomethane. Each measurement was done 3 times using a 5 µl drop of the testing liquid to avoid gravity effects. Images were processed in ImageJ[2] using the contact angle plugin. To validate the accuracy of this method, the values obtained were compared to those reported in the literature. The values estimated using the geometric mean for graphene oxide (GO) and thermally reduced graphene oxide (RGO) were 61.7 and 44.6 mJ/m^2, respectively. These are in good agreement with those reported previously by Wang *et al.,* reported as 62.1 and 46.7 mJ/m^2 for GO and RGO, respectively.[3] Therefore, these values, along with polar and dispersive parts (γ^d and γ^p) were used to estimate the preferential localization of the graphene-based particles before reacting with PLA. The wetting coefficient ω is given by:

$$\omega = \frac{\gamma_{PS/G} - \gamma_{PLA/G}}{\gamma_{PS/PLA}} \qquad \text{Eq. S1}$$

Table S2. Estimations of the reactive graphene localisation-based on wetting coefficient calculations.

	Surface energy $\gamma_s = \gamma_p + \gamma_d$ $[mJ/m^2]$	γ_p $[mJ/m^2]$	γ_d $[mJ/m^2]$	Reference
Polystyrene (PS)	42.6	1.4	41.2	[4]
Polylactic acid (PLA)	43.5	3.9	39.6	[5,6]
Reactive graphene	46.4	10.5	35.9	

Geometric mean estimation	
Wetting coefficient	Localisation
4.2	PLA phase

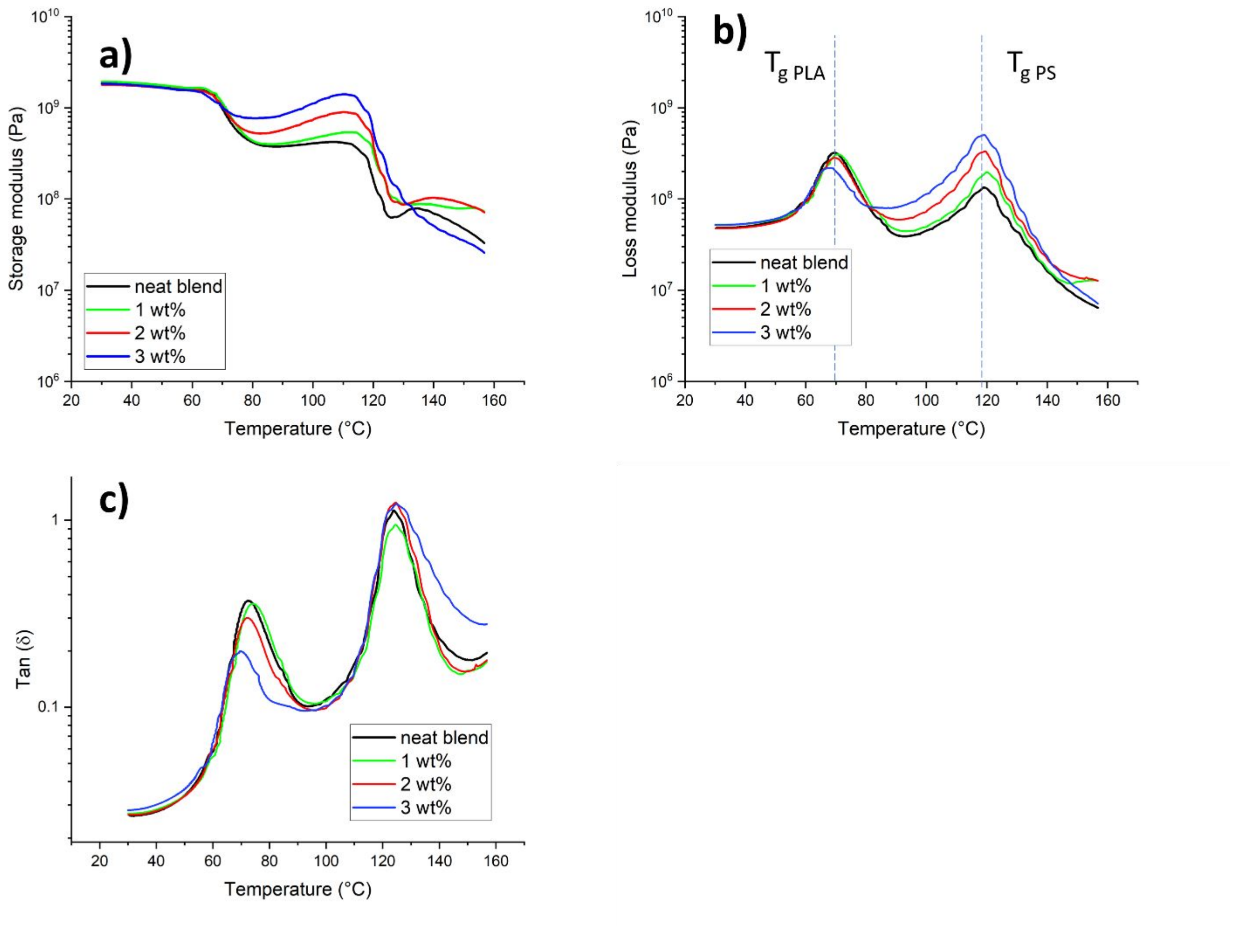

Figure S1 DMA data to show a) Storage modulus, b) loss modulus and c) tan δ graphs of the neat blend and blends containing reactive graphene. Storage modulus shows higher magnitudes at the plateau region (80 - 110 °C) with increasing the reactive graphene content from 1 to 3 wt%. Since reactive graphene was premixed with PLA and only interacts with the PS phase through the interface, it suggests a compatibilization effect due to the interfacial localisation of graphene.

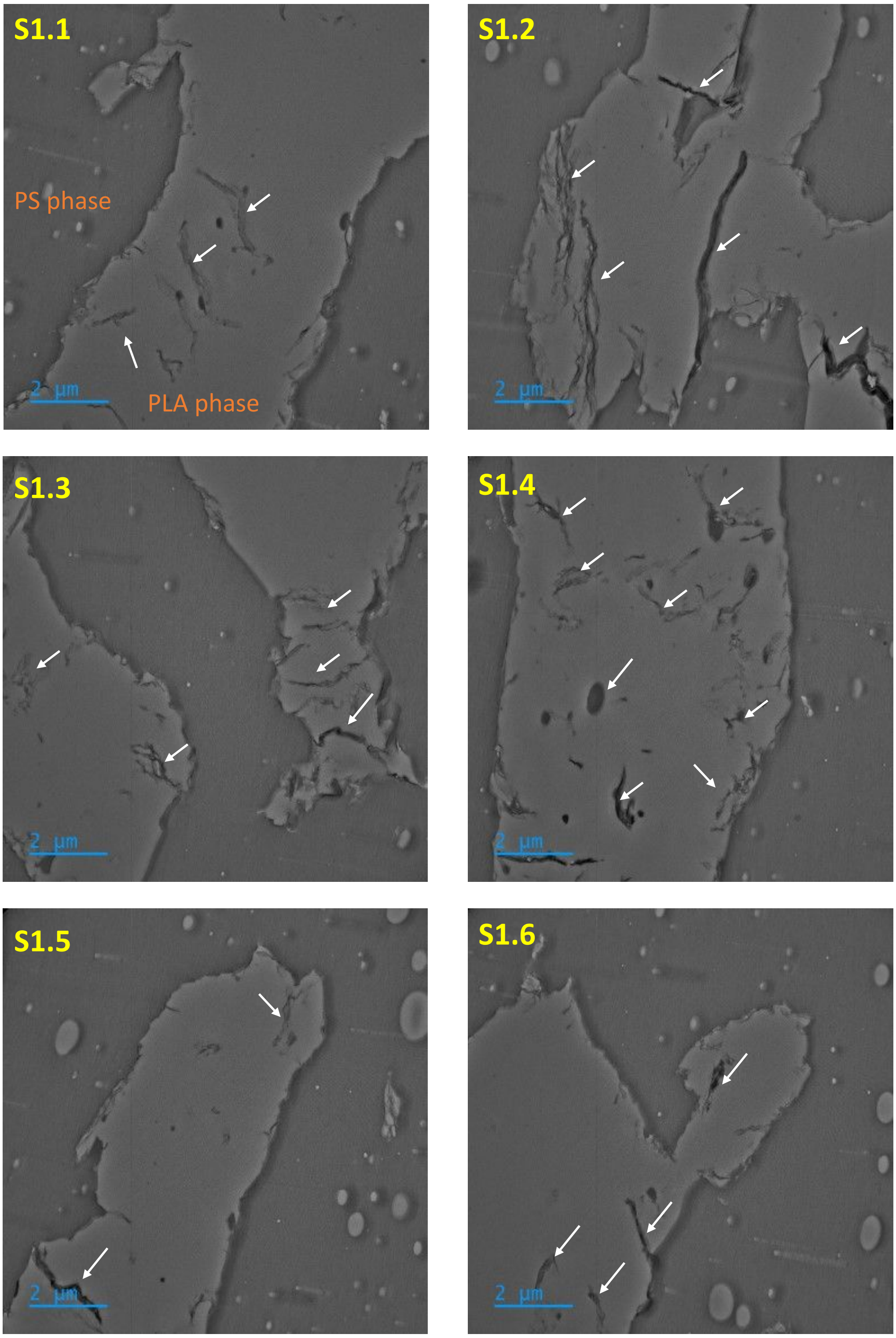

Figure S2 TEM images from selected areas showing the reactive graphene localized at the PLA phase (pointed out with white arrows) and at the interface.

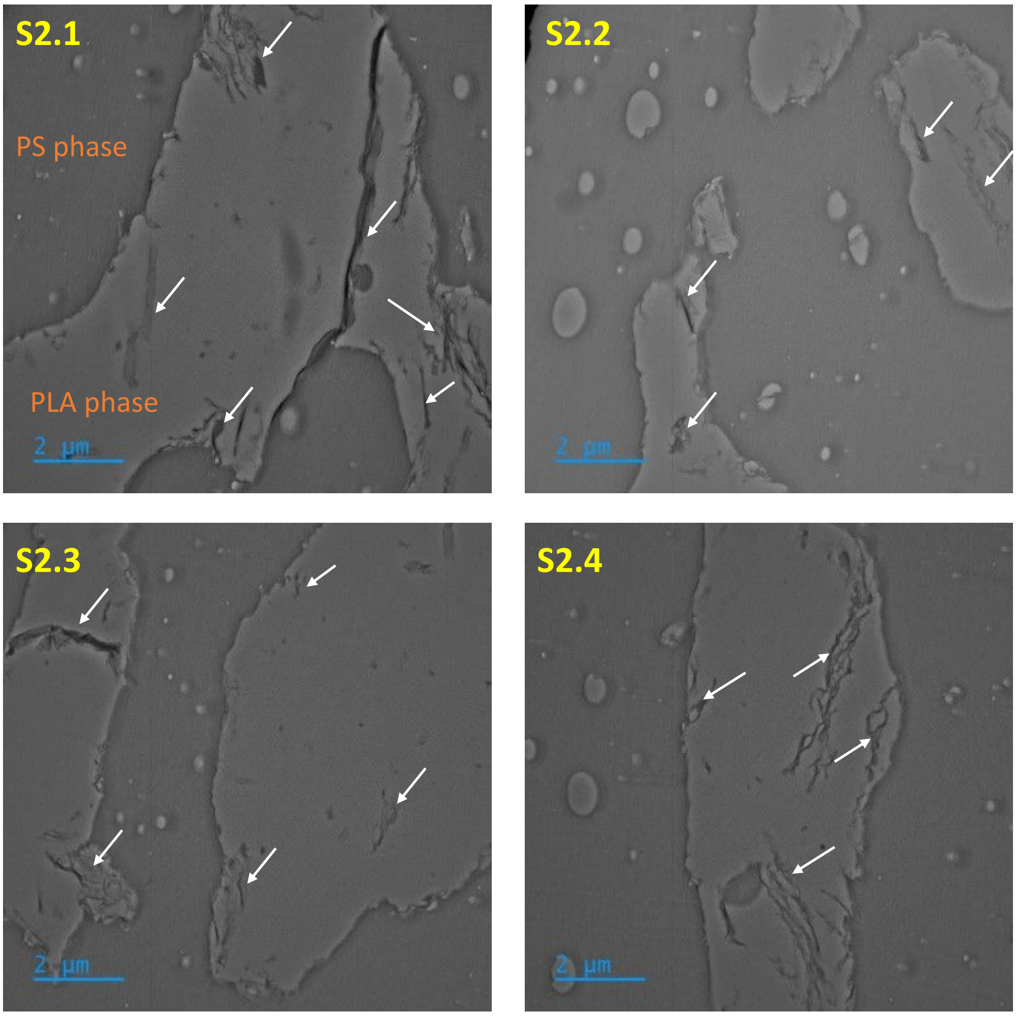

Figure S3 TEM images from selected areas showing the reactive graphene localized at the PLA phase (pointed out with white arrows) and at the interface.

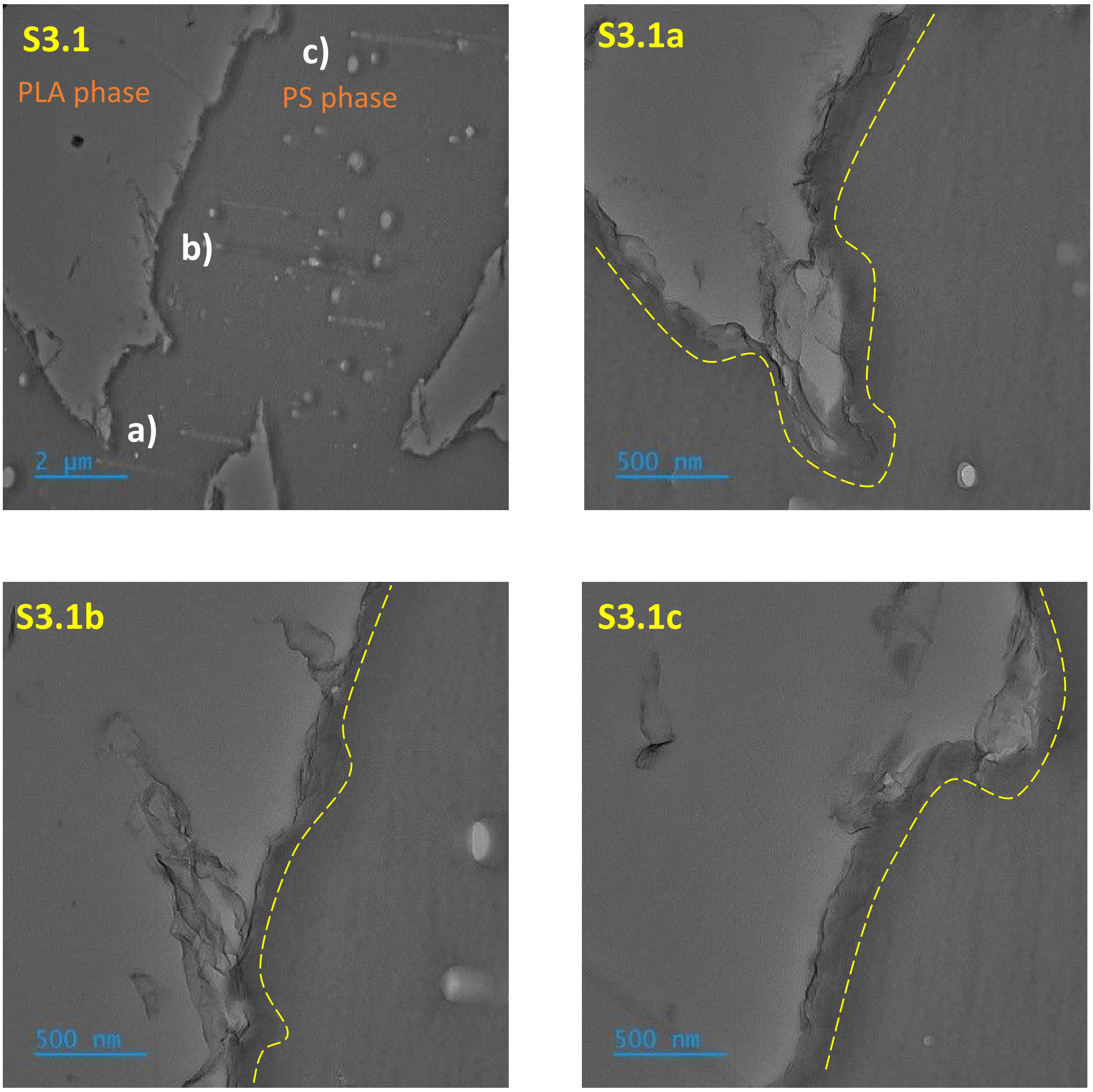

Figure S4 High magnification TEM images from selected areas in Figure S3.1 showing the reactive graphene localized at the PS/PLA interface. Dashed yellow lines highlight the sections where graphene is located.

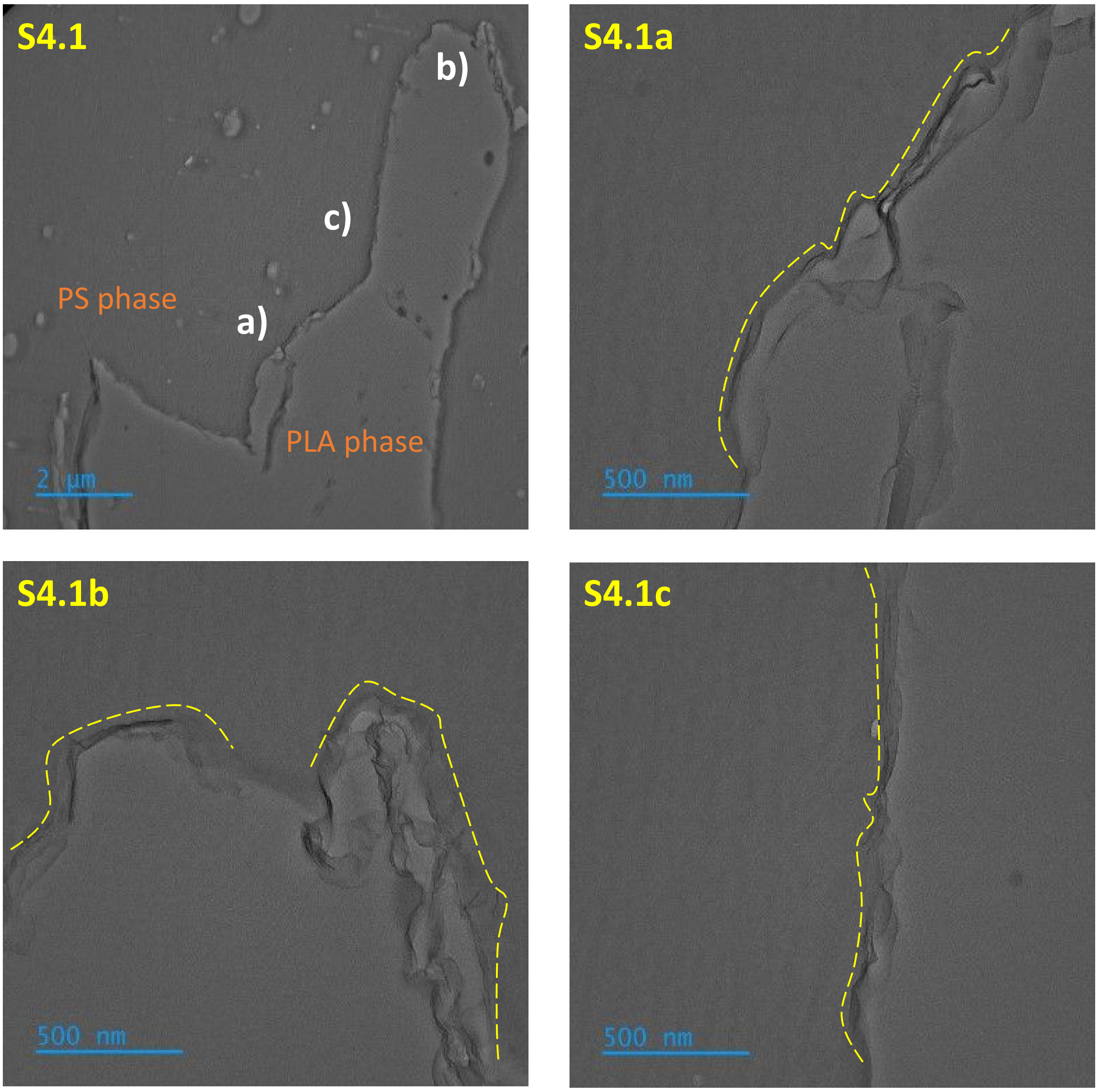

Figure S5 High magnification TEM images from selected areas in Figure S3.1 showing the reactive graphene localized at the PS/PLA interface. Dashed yellow lines highlight the sections where graphene is located.

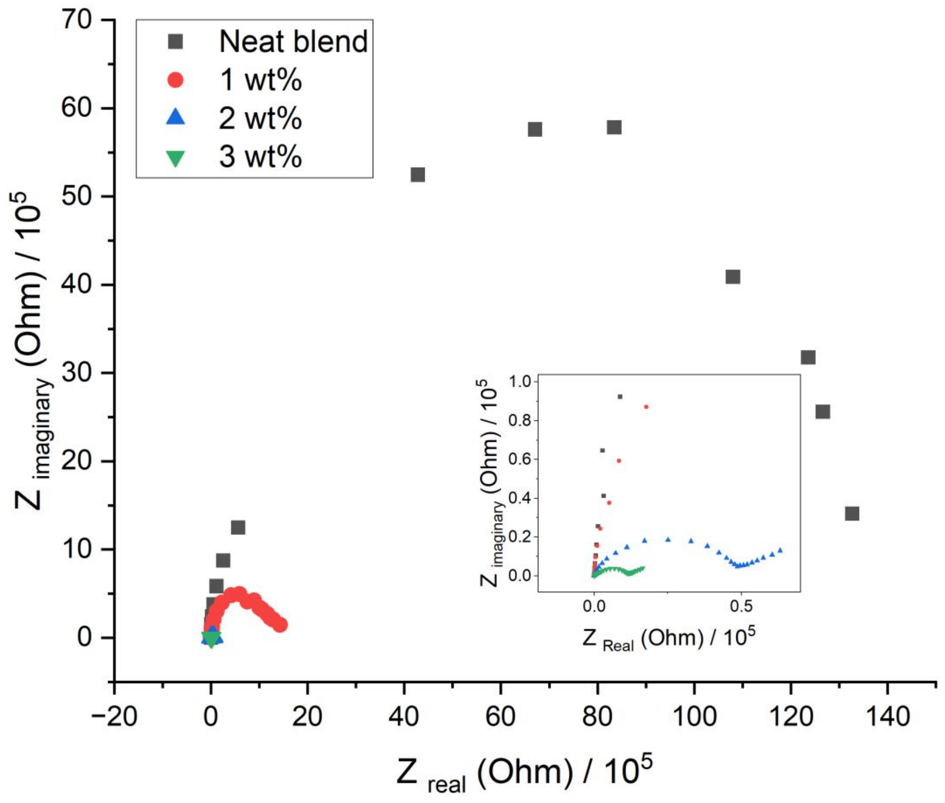

Figure S6 Nyquist plots based on the data presented in Figure 11, considering the effect of different loadings of reactive graphene in the PLA/PS composite (from 1 to 3 wt%) and the neat blend.

References

(1) Bhattacharjee, S. DLS and Zeta Potential – What They Are and What They Are Not? *J. Control. Release* **2016**, *235*, 337–351. https://doi.org/10.1016/j.jconrel.2016.06.017.

(2) Schneider, C. A.; Rasband, W. S.; Eliceiri, K. W. NIH Image to ImageJ: 25 Years of Image Analysis. *Nat. Methods* **2012**, *9*, 671–675. https://doi.org/10.1038/nmeth.2089.

(3) Wang, S.; Zhang, Y.; Abidi, N.; Cabrales, L. Wettability and Surface Free Energy of Graphene Films. *Langmuir* **2009**, *25*, 11078–11081. https://doi.org/10.1021/la901402f.

(4) Wu, S. Chapter 3. Interfacial and Surface Tensions of Polymer Melts and Liquids. In

Polymer Interface and Adhesion; CRC Press, Ed.; Marcel Dekker: New York, 1982; pp 133–168.

(5) Ringard-Lefebvre, C.; Baszkin, A. Behavior of Poly (D, L-Lactic Acid) Monolayers at the Air-Water Interface. Effect of Spreading Solvents. *Langmuir* **1994**, *10*, 2376–2381. https://doi.org/10.1021/la00019a057.

(6) Biresaw, G.; Carriere, C. J. Interfacial Tension of Poly(Lactic Acid)/Polystyrene Blends. *J. Polym. Sci. Part B Polym. Phys.* **2002**, *40*, 2248–2258. https://doi.org/10.1002/polb.10290.
